# H3K27me3 in Diffuse Midline Glioma and Epithelial Ovarian Cancer: Opposing Epigenetic Changes Leading to the Same Poor Outcomes

**DOI:** 10.3390/cells11213376

**Published:** 2022-10-26

**Authors:** Charles A. Day, Edward H. Hinchcliffe, James P. Robinson

**Affiliations:** 1 Hormel Institute, University of Minnesota, Austin, MN 55912, USA; 2 Mayo Clinic, Rochester, MN 55902, USA; 3 Masonic Cancer Center, University of Minnesota, Minneapolis, MN 55455, USA

**Keywords:** diffuse midline glioma, diffuse intrinsic pontine glioma, epithelial ovarian cancer, epigenetics, histone H3, H3K27M, K3K27me3, EZH2, PRC2

## Abstract

Histone post-translational modifications modulate gene expression through epigenetic gene regulation. The core histone H3 family members, H3.1, H3.2, and H3.3, play a central role in epigenetics. H3 histones can acquire many post-translational modifications, including the trimethylation of H3K27 (H3K27me3), which represses transcription. Triple methylation of H3K27 is performed by the histone methyltransferase Enhancer of Zeste Homologue 2 (EZH2), a component of the Polycomb Repressive Complex 2. Both global increases and decreases in H3K27me3 have been implicated in a wide range of cancer types. Here, we explore how opposing changes in H3K27me3 contribute to cancer by highlighting its role in two vastly different cancer types; (1) a form of glioma known as diffuse midline glioma H3K27-altered and (2) epithelial ovarian cancer. These two cancers vary widely in the age of onset, sex, associated mutations, and cell and organ type. However, both diffuse midline glioma and ovarian cancer have dysregulation of H3K27 methylation, triggering changes to the cancer cell transcriptome. In diffuse midline glioma, the loss of H3K27 methylation is a primary driving factor in tumorigenesis that promotes glial cell stemness and silences tumor suppressor genes. Conversely, hypermethylation of H3K27 occurs in late-stage epithelial ovarian cancer, which promotes tumor vascularization and tumor cell migration. By using each cancer type as a case study, this review emphasizes the importance of H3K27me3 in cancer while demonstrating that the mechanisms of histone H3 modification and subsequent gene expression changes are not a one-size-fits-all across cancer types.

## 1. H3K27me3 Involvement in Epigenetic Gene Regulation

Epigenetics are mechanisms of gene transcriptional regulation in response to specific modifications to either DNA or histones. The histone superfamily is divided into the core histones (H2A, H2B, H3, and H4) and the linker histones (H1 and H5). The core histones assemble into nucleosomes containing two copies of H2A, H2B, H3, and H4 wrapped by 147 bp of DNA. Linker histones sit as monomers on the DNA between the nucleosomes. The core histones share a basic structure comprised of a core domain and a flexible n-terminal tail domain. The core domain facilitates the binding to other histones in the nucleosome and to the DNA, thus allowing for the packaging of the genome into the nucleus. The n-terminal tails of the core histones are exposed to the nucleoplasm and can acquire a variety of post-translational modifications (PTMs). Histone PTM’s alter the chromatin accessibility and binding affinity by various proteins, including transcriptional co-factors. In these ways histone PTMs can influence gene transcription.

Some H3 histone family members play important roles in epigenetic gene regulation. In humans, the H3 family is comprised of eight members: H3.1, H3.2, H3.3, H3.4 (also known as H3t), H3.5 (also known as H3.3C), H3X, H3Y, and CENPA (reviewed in [1,2]). CENPA is centromere specific and has no direct role in gene expression. H3.4, H3.5, H3X, and H3Y are very tissue-specific, and their functions are poorly characterized [3,4,5,6,7]. The final three H3 proteins, H3.1, H3.2, and H3.3, are expressed throughout the human body. These three central H3 family members are well characterized and play prominent roles in gene regulation.

The H3 family has extreme genetic redundancy with ten genes for H3.1 (*HIST1H3A*-*HIST1H3J*), three genes for H3.2 (*HIST2H3A*, *HIST2H3C*, and *HIST2H3D*), and two genes encoding H3.3 (*H3F3A* and *H3F3B*). The first amino acid on the H3 histone tail is cleaved after the translation of these proteins. The convention, therefore, is to name H3 residues starting from the second encoded amino acid [8]. Thus, K27 is technically the 28th amino acid encoded by the H3 genes. The three classical H3 proteins show remarkable amino acid similarity as H3.1 and H3.2 differ at only one amino acid residue (position 96). Furthermore, H3.3 differs from H3.1 by five residues (positions 31, 87, 89, 90, and 96) [9]. Of the residues that differ between H3.1/H3.2 and H3.3, only residue 31 (alanine in H3.1 and H3.2, serine in H3.3) is in the n-terminal tail of the protein with the remaining differential residues (between cites 87 and 96) in the histone core.

The unique amino acids in the core domains of these histones create distinct binding domains for specific histone chaperone proteins. H3.1 and H3.2 are loaded by CAF-1 along the chromosome arms during S phase [10,11]. Histone H3.3 is a replication-independent variant that is expressed throughout interphase [12]. H3.3 has its own histone chaperones that insert the H3 proteins into distinct regions of the chromosome. The chaperone HIRA loads H3.3 along the histone arms [11]. H3.3 is also highly enriched at the pericentromere and telomeres. Loading at these sites is not facilitated by HIRA but by a complex of ATRX and DAXX [13].

H3.3 is highly enriched along the chromosome arms at transcription start sites and regulatory regions such as gene promoters and enhancers [13]. If wild-type H3.1 and H3.2 are also enriched at specific genomic elements is unclear. However, work with mutant H3.1 and H3.3 variants suggests while H3.3 is enriched at transcriptional start sites, H3.1 may be uniformly dispersed across the genome [14].

Histone tails can be post-translationally modified by ubiquitination, phosphorylation, acetylation, mono-, di-, or tri-methylation. Acetylation and methylation of lysines are opposing modifications and often function in epigenetic gene regulation. Lysine 27 on H3 can be acetylated, mono-, di- or tri-methylated (H3K27ac, H3K27me1, H3K27me2 and H3K27me3, respectively). Importantly, H3K27me3 is associated with the repression of gene transcription, while H3K27 acetylation (H3K27ac) is linked to gene expression.

H3K27 methylations are performed by the Polycomb Repressive Complexes 2.1 and PRC2.2, collectively termed PRC2. The PRC2 complexes share the structural subunits; Suz12, EED, and RBBP4/7 (also known as RbAp48/46). Where they differ is in the variety of accessory complex units. PRC2.1 contains the accessory subunits PCL1-3, EPOP, and PALI1/2, while PRC2.2 contains AEBP2 and JARID2. The Suz12 and EED subunits of the core PRC2 complex, and JARID in PRC2.2, recognize the H3 tail for complex binding [15,16,17]. Additionally, both complexes can contain either the methyltransferase Enhancer of Zeste Homolog 1 (EZH1) and PRC2 containing Enhancer of Zeste Homolog 2 (EZH2, also called KMT6A) as their enzymatic subunit.

The primary role of PRC2 is to deposit the K27 methylations on H3. EZH2 containing PRC2 complexes place primarily the H3K27me2 and H3K27me3 marks. Furthermore, while EZH2 can perform H3K27me1, this activity is much weaker than its di- and tri-methylation activity [18,19]. PRC2 complexes containing EZH1 have a generally lower activity than PRC2/EZH2 for K27 di- and tri-methylation. Instead, EZH1 primarily performs K27 mono-methylation [19,20]. Interestingly, PRC2 complexes can also automethylate EZH2 and SUZ12 [21,22,23]. This automethylation boosts the intrinsic activity of PRC2 to methylate H3K27 [23]. Importantly, the PRC2 complex has little or no methyltransferase activity for other histone residues [16,24].

A related complex to PRC2 is PRC1. There are a variety of PRC1 subcomplexes (reviewed in [25]), but all contain the ubiquitin E3 ligase, RING1A/B, and one of six Polycomb RING finger proteins [26]. Unlike PRC2, which performs methylation, PRC1 performs H2AK119 ubiquitination (H2AK119ub) through its enzymatic subunit, RING1A/B [27]. PRC1 and PRC2 can work in a feedback mechanism to retain H3K27me3. Here, H2AK119ub recruits PRC2.2 via recognition by the JARID2 subunit [17,27,28], leading to the trimethylation of H3K27. Furthermore, H3K27me3 serves as a docking site for PRC1 [16,24].

Demethylation of H3K27 is performed primarily by KDM6A (UTX) and KDM6B (JMJD3) [29,30]. KDM6A is encoded on the X chromosomes, and a very similar gene to *KDM6A*, known as *UTY*, exists on the Y chromosome [31]. *UTY* encodes KDM6C (or UTY), which can also demethylate H3K27. However, the K27 demethylase activity of KDM6C is dramatically reduced compared to KDM6A or KDM6B [32].

H3K27 triple methylation plays a significant role in cell differentiation. Multiple studies have shown that impairment of PRC2 leads to stalled differentiation of embryonic stem cells [19,33]. In one such study, ChIP-on-ChIP using antibodies for H3K27me3 and PRC2 components was performed on human embryonic stem cells [34]. They reported an enrichment PRC2 and H3K27me3 of genes involved in neuronal differentiation, bone differentiation, sex differentiation, muscle development, and hematopoiesis. When the experiment was repeated 2, 5, and 10 days after initiating neuronal differentiation, the H3K27me3 levels dropped dramatically at some genes, such as *HOXA9-13* [34]. At the same time, some of the H3K27me3 marked genes showed an increase in H3K27me3 during differentiation. This heightened repression of transcription was observed in well-known developmental genes such as *NEUROG2*, *OLIG2*, and *HOXA1-5* [34].

H3.3K27me3 often functions in concert with the H3K4me3 mark; at what are known as “poised” or “bivalent” sites (i.e., sites where expression is silenced but ready to be activated) [35]. H3K4me3 is an activating mark for transcription and is placed by a Thithorax complex comprised of WDR5, RBBP5, ASH2L, DPY-30, and an enzymatic subunit. A variety of proteins can serve as the enzymatic subunit of this complex, including MLL1-4, SETD1A, and SETD1B [36]. Furthermore, H3K4me3 is demethylated by KDM2B (also known as JHDM1B/FBXL10) [37]. Poised promotors containing the H3K4me3/K27me3 double mark generally show very low-level expression, which can quickly be up- or down-regulated with the loss of K27me3 or K4me3, respectively. Poised enhancers play a central role in stem cell differentiation as genes are turned on or off during lineage maturation [38,39]. Recent work has shown that H2AK119ub is also found at many poised promoters, and whether it is silencing associated genes or recruiting PRC2 to methylate H3K27 is uncertain [40].

In addition to its prominent role in development, changes in H3K27me3 have been observed in many cancer types (Table 1), where they play complex roles in all stages of cancer formation, maturation, and metastasis. For example, overexpression of wild-type (WT) EZH2, and subsequent increases in H3K27me3, have been described in several non-hematological cancers, including prostate and breast cancer [41,42,43,44], where evidence suggests that the increased H3K27me3 levels promote tumor progression and metastasis. EZH2 mutations have also been observed in some cancers, such as B- and T-cell lymphoproliferative disorders [45].

**Table 1:** List of cancer types with reported changes in H3K27me3 levels.

Cancer Type	H3K27me3 Change	Cause of K27me3 Change
Bladder	decrease [46]	EZH2 overexpression [47,48]
Breast	increase [49]	EZH2 overexpression [41,42,43]
Central nervous system		
Ependymoma	decrease [50,51]	EZHIP overexpression [52]
Meningioma	decrease [53,54,55]	unknown
Oligodendroglioma	decrease [56]	unknown
Diffuse midline glioma	decrease [57,58,59,60]	H3 K27M mutation [61,62], EZHIP overexpression [59,60]
Peripheral nerve sheath	decrease [63,64]	EED & SUZ12 loss-of-function mutations [63]
Colo-rectal	unknown	EZH2, EED, SUZ12 overexpression [65]
Endometrial	increase [66]	EZH2 overexpression [67]
Esophageal	increase [68,69]	EZH2 overexpression [70]
Gastric	increase [71,72]	EZH2 overexpression [73]
Leukemia		
T-cell acute lymphoblastic	decrease [74]	EZH2 loss-of-function mutation [74]
Liver	increase [75]	EZH2 overexpression [76]
Lung		
Non-small cell	increase [77]	EZH2 overexpression [78,79]
Small cell	increase [80]	EZH2 overexpression [80]
Lymphoma		
Hodgkins	unreported	KDM6B overexpression [81]
Non-Hodgkins		
B-Cell	increase [45,82,83]	EZH2 gain-of-function mutation [45]
Diffuse large B-Cell	unreported	KDM6B overexpression [84,85]
Natural Kill/T-cell	increase [86]	EZH2, EED, SUZ12 overexpression [86]
Melanoma	increase [87]	EZH2 overexpression [87,88,89], EZH2 gain-of-function mutation [90]
Myeloproliferative neoplasm/Myelofibrosis	decrease [91]	EZH2 loss-of-function mutation [91]
Ovarian Cancer	increase [92]	EZH2 overexpression [92,93]
Pancreatic	decrease [94]	EZH2 overexpression [95]
Prostate	increase [96]	EZH2 overexpression [44]
Renal	unreported	EZH2 overexpression [97,98]

## 2. H3K27me3 in Pediatric Diffuse Midline Glioma

### 2.1. Epidemiology of Pediatric Diffuse Midline Glioma

**Figure 1:**
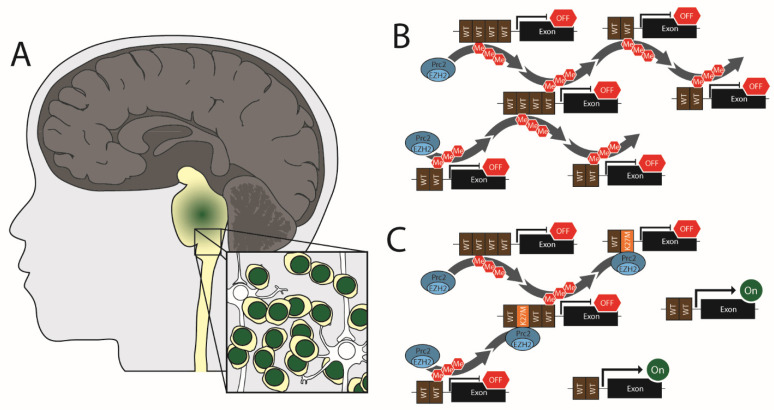
Pediatric Diffuse Midline Glioma. (**A**) Diffuse midline gliomas arise primarily in the pons of the brain stem. (**B**) Normal cells have mobile PRC2 complex that facilitates H3K27 triple methylation on promoter regions leading to the silencing of genes. (**C**) In DMG H3K27 altered cells, PRC2 complexes become trapped at H3 K27M loci leading to a reduced pool of active PRC2 and subsequent activation of genes.

In pediatrics, high-grade gliomas can occur in the cerebral hemispheres but are most common in the midline (i.e., the spinal cord, thalamus, and brainstem) (Figure 1). The current WHO guidelines classify these midline tumors as diffuse midline gliomas (DMG) [99]. Because these tumors arise primarily from the pons, they have historically been referred to as diffuse intrinsic pontine glioma (or DIPG). Therefore, both DMG and DIPG nomenclatures are common in the literature. DMGs do occur in adults and adolescents but are most prevalent in pediatric patients [100,101,102,103]. Approximately 300 children are diagnosed with diffuse midline gliomas in the United States annually [104], with cases roughly equally distributed between males and females [105]. These are the most aggressive form of childhood cancer, with a median survival of 9–11 months and a 5-year survival rate of ~2 percent [106].

As the name “Diffuse Midline Glioma” suggests, these tumors are highly diffuse and intercalated within the surrounding brain tissue. A few case reports have noted metastatic diffuse midline gliomas [107,108,109], but this occurrence is extremely rare. The highly diffuse nature and the location of these tumors in or near the brain stem often prevents radiotherapy or full surgical redaction. Furthermore, targeting these tumors with chemotherapy has not proven effective. The ability of the blood–brain barrier to prevent expel small molecules from the brain is well known. Furthermore, diffuse midline gliomas often have intact tumor vasculature, as evidenced by the lack of contrast enhancement in magnetic resonance imaging [110]. The appearance of an intact blood–brain barrier is supported by the histology of DMG tumors in animal models, which show normal vasculature morphology and extravascular permeability [111]. This intact vasculature accounts, at least in part, for the lack of effective small molecule treatments for this tumor type.

Point mutations in H3 have been identified as early events for up to 40% of pediatric gliomas [61,62]. These mutations in the H3 proteins occur only at amino acids K27 and G34. At K27, the mutation is almost exclusively K27M. This mutation is found in as many as 80% of diffuse midline gliomas [61,62]. An extremely rare H3K27I mutation has also been reported [112] and probably contributes to tumorigenesis through the same mechanisms as the K27M mutation [112,113]. H3 K27M is an acquired somatic mutation that occurs in only a signal allele of H3.1, H3.2, or H3.3. The prevalence of the K27M mutation in these genes is not equal, with ~20% of diffuse midline gliomas having H3.1 K27M mutations vs. ~60% with H3.3 K27M mutations [62]. Only a single case of H3.2 K27M has been reported [112]. Studies of pediatric glioma have found dramatic differences in prognostic outcomes among patients depending on their H3 status. H3.3 K27M has the worst outcome of all pediatric gliomas, with median overall survival of 9.2 months post-diagnosis. In comparison, H3.1 K27M patients survive significantly longer, with median overall survival of 15.0 months [112].

A second set of H3 mutations, H3 G34R/V, have also been identified in diffuse glioma [61,62]. Interestingly, the tumors associated with mutations at K27 and G34 are distinct in presentation and underlying biology. For example, the H3 G34R/V tumors are primarily found in the brain hemispheres, not the brain stem [61,114]. Notably, the H3 G34R/V mutation has little, if any, effect on post-translational modifications at K27 [113,115]. Rather H3 G34R/V causes reduced trimethylation at H3K36 of the mutant H3 mutant protein [113,116,117], a mark generally considered activating for transcription [118]. This leads to different gene expression changes in H3 G34-mutant tumors than in H3 K27M tumors [61,114,117,119,120]. Furthermore, patients with H3 G34R/V mutant tumors have better long-term survival than those with H3 K27M mutant tumors [114,117,120]. This difference in survival does not appear to be linked to tumor location, as the difference in survival between mutation types persists even when the tumor location is the same [120]. The discrepancy between K27M and G34R/V patient outcomes is not apparent, though it may result from different gene expression [61,114,117,119]. Finally, these tumors have abnormal tumor vasculature [111] which may support the hypothesis that small molecule delivery to these tumors is more effective. While H3 K27M and H3 G34R/V tumors were for a long time grouped as one cancer type, the 2021 WHO Classification of CNS Tumors divided them into distinct types; “diffuse midline glioma, H3 K27-altered” and “diffuse hemispheric glioma, H3 G34-mutant [99].”

A third class, “diffuse pediatric-type high-grade glioma, H3-wildtype, and IDH-wildtype,” was also established in recent years. These tumors were previously grouped under diffuse intrinsic pontine glioma with H3 K27M and G34R/V tumors. However, as they lack any H3 mutation and have normal H3K27me3 levels, among other unique features, they were separated from the H3K27-altered and G34-mutant tumors by the World Health Organization in 2021 [99]. The separation of pediatric diffuse high-grade glioma into three distinct tumor types emphasizes the importance of histological staining in diagnosing diffuse glioma.

### 2.2. Altered H3K27me3 Levels in Diffuse Midline Glioma, H3 K27-Altered

Shortly after H3 mutations were identified as glioma drivers, multiple groups reported a near total loss of H3K27me3 staining, by immunohistochemistry and Western blotting, in H3 K27M positive diffuse midline gliomas [57,58]. As H3 K27M accounts for between 3.6% and 17.6% of total H3 protein in human diffuse midline gliomas tumor cells [113], the observed decline in K27me3 could not be attributed to a fraction of the total H3 no longer containing a Lysine at residue 27. Furthermore, overexpression of H3.1 K27M or H3.3 K27M in H3 WT cells showed this same loss of K27me3, demonstrating that it is a direct result of the H3 mutation and not the result of a co-occurring mutation [57]. The observed changes in H3K27me3 could also not be directly attributed to changes in the activity of methylation enzymes, as EZH2 expression levels are normal in the H3 K27M tumors [58] and patient-derived cell lines [57]. Furthermore, no mutations in EZH2 have been found in pediatric glioma [61,114,121]. Likewise, a gain of demethylation activity could not explain the observed global loss of K27me3 since activity of the K27me3 demethylases, KDM6A and KDM6B, were similar in H3 K27M and H3 WT expressing cells [122].

To address why global H3K27me3 was lost, Lewis et al. introduced transgenic H3.3 plasmids encoding each of the 20 amino acids at residue 27 into 293T cells. Using blotting, they found that only H3.3 K27M and H3.3 K27I mutations could induce global loss of H3K27me3 [113]. In a separate study, Bender et al. used immunoprecipitation from multiple human cell lines to reveal that the PRC2 component, SUZ12, has a much higher binding affinity for H3.3 K27M than for WT H3.3 [122]. These findings have led to a popular model where H3.3 K27M binds and traps the PRC2 complex, thus restricting it from performing methylation at other sites (Figure 1). More recently, it has been shown that H3 K27M mutation also inhibits the automethylation of EZH2 by PRC2, suggesting that H3 K27M could drive global loss of H3K27me3 by suppressing PRC2 activation [123].

Notably, H3 K27M has only minor effects on H3K27me1 levels [124]. Additionally, methylation of other H3 residues is unaffected by the K27M mutation [57,113,122]. Furthermore, the loss of H3K27me3 globally leads to an increase in the activating K27ac mark [113,125,126]. Very little research has been done into H2AK119ub levels in K27M mutant tumors. However, one recent study found that K27M expression stimulates the expression of the PRC1 subunit, BMI1, leading to higher H2AK119ub in cells [127].

Interestingly, ~4% of diffuse midline gliomas have no H3 mutations but still exhibit loss of H3K27me3 [60]. This loss of H3K27me3 results from the overexpression of Enhancer of Zest Homologs Inhibitory Protein or EZHIP (also known as CXorf67) [59,60]. EZHIP probably has no enzymatic activity of its own but binds to EZH2 and SUZ12 while also having a low affinity for EZH1 [128,129]. EZHIP specifically binds PRC2 on chromatin, reducing its mobility and triggering global H3K27me2/3 loss [128,129,130]–reminiscent of the proposed model for EZH2 suppression by H3 K27M trapping.

### 2.3. Transcriptional Changes Linked to Loss of H3K27me3 in Diffuse Midline Glioma

The global loss of H3K27me3 causes large-scale changes to the cell transcriptome. Molecular profiling has identified thousands of genes that lose H3K27me3, thus becoming transcriptionally upregulated in K27M tumors. For example, when CRISPR was used to revert the H3 K27M allele to WT in two separate DIPG patient lines, ChIP-Seq of the isogenically paired lines found significant changes in H3K27me3 at 97,768 and 32,239 loci in the two paired cell lines [131]. Of the thousands of effected gene there has been little consensus over which affected genes are of primary importance for diffuse midline glioma formation, however, some broad trends have emerged. Bioinformatic analysis of RNA-Seq and ChIP-Seq data from K27M cells has repeatedly found neurodevelopment, synapse formation/signaling and glial cell differentiation genes as the most differentially transcribed genes [57,119,122,125,131,132,133]. In one such study, Bender et al. compared gene expression between twelve H3 K27M and ten H3 WT pediatric gliomas and found 294 differentially expressed genes that largely coincided with gene loci where K27me3 changes were observed [122]. Subsequent gene ontology (GO) analysis of this data set found that the differentially expressed genes were primarily involved in neuronal differentiation [122]. Similarly, GO analysis of H3.3 K27M vs. H3.3 WT mice found neurodevelopment genes and glial identity genes as the most upregulated groups [125,133].

Single-cell RNA-Seq of tumor cells from 6 patients with H3 K27M diffuse midline gliomas revealed that the tumor cell transcriptome most closely resembled Oligodendrocyte Precursor Cells (OPC)–a stem-like cell with a high capacity for self-renewal and a precursor to oligodendrocytes and astrocytes development [134]. This finding, along with the identification of neuro-development genes in the cluster analysis, has led to the idea that H3 K27M either appears in these OPC-like cells or reprograms a glial cell back to the OPC-like state. This progenitor cell then initiates tumor formation while maintaining a highly plastic and proliferative stem-like tumor cell population.

Signaling pathways that promote cell growth are also affected at the epigenetic level in H3K27-altered tumors. Elevated expression of platelet-derived growth factor receptor A (PDGFRA) is a common feature of pediatric diffuse midline glioma [135,136]. This can result from either *PDGFRA* amplification, activating mutation, or the loss of the repressive K27me3 at the *PDGFRA* gene [61,122,135]. Notably, the OPC-like cancer cell population in K27M tumors is highly dependent on PDGF signaling [134]. This can lead to a feedback loop where K27M stimulates PDGF signaling, which feeds the growth of H3 K27M tumor cells.

Other proliferative pathways are also transcriptionally upregulated in H3 K27M positive diffuse midline gliomas. First, analysis of paired K27M lines with cells where the K27 was reverted to WT by CRISPR found the K27M mutation associated with loss of K27me3 at multiple super-enhancers for NOTCH signaling genes, including *ASCL1*, *HES5*, and *NOTCH*. Further analysis by RNA-Seq and RT-PCR confirmed an upregulation in NOTCH signaling proteins due to the K27M mutation [131]. Second, MYC and MYCN levels also increase in response to K27M mutagenesis [131]. MYC is a well-known oncogene that regulates the expression of many proliferative genes, and its overexpression has been shown to promote glioma formation in mice [137].

### 2.4. Selective Retention of H3K27me3 in Diffuse Midline Glioma

While H3K27me3 is depleted globally in H3 K27M cells, K27me3 levels are retained (or even elevated) at a small number of loci in H3 K27M human tumors, patient-derived cell lines, transgenic cell lines, and animal models [57,122,125,126,132,138,139]. For example, one analysis found that 6% of the H3K27me3 sites identified in WT cells had higher levels of H3K27me3 in H3.3 K27M tumor samples [140]. ChIP-Seq of mouse neuronal stem cells overexpressing H3.3 WT or H3.3 K27M identified two interesting trends that may explain this phenomenon. First, H3 is enriched in regions with abundant CpG dinucleotide (known as CpG islands or CGI’s) along the chromosome arms. CpG islands with the highest H3K27me3 levels in WT cells were the most likely to retain K27me3 in K27M cells [126]. This agrees with a later finding that PRC2 has a high binding affinity for H3 that is bound to unmethylated CGI’s [124]. The high binding affinity for CGI’s supports a model where free PRC2 (not captured by K27M) is more likely to accumulate at unmethylated CGI’s and methylate the associated H3 proteins than at other regions of the genome. Second, Mohammed et al. observed that the more physically isolated a CGI is in the genome, the more likely it is to have lost K27me3 in K27M cells. Alternatively, those CGI’s that are physically near other CGI’s or are part of exceptionally long CpG sequences are more likely to retain K27me3 [126]. Interestingly, there was a near total overlap in the genes that retained or gained H3K27me3 when compared across H3.1 K27M and H3.3 K27M patient cell lines, indicating that the continued silencing of these genes may be of particular importance to glioma formation and growth [140].

The retention of H3K27me3 at specific sites is highly beneficial to diffuse midline glioma K27-altered tumors as it silences the expression of some essential tumor suppressor genes, including those involved in cell cycle arrest. ChIP-Seq and RNA-Seq studies revealed that the p16 gene, *CDKn2a*, is highly repressed in H3 K27M patient-derived lines [57,126]. *CDKn2a* was also among the genes most strongly repressed by K27me3 in the presence of H3 K27M in mouse cells [126,132] and p16 expression was downregulation in a mouse H3 K27M tumor model [133]. p16 outcompetes Cyclin D for binding to CDK6, leading to a blockage for cell cycle progression into S phase; thus, the loss of p16 means a lack of checkpoint control at the G1-S interface. The G1-S transition appears to be a hotspot for dysregulation in these tumors as K27me3 levels also increase at the long-form gene for CDK6, leading to a loss of CDK6 expression in H3 K27M cells [57,122].

Besides cell cycle genes, H3K27me3 is retained at other key tumor suppressor genes. For instance, the H3K27me3 mark is also retained at the gene for Wilms Tumor 1 (*WT1*) [140]. WT1 is a zinc finger transcription factor linked to tumor suppression [141] and oncogenic functions in various brain cancer types [142,143,144,145]. In diffuse midline glioma K27-altered tumors, WT1 appears to have tumor suppressor activity as transient overexpression of WT1 or Cas9 targeted removal of the repressive H3K27me3 mark on the WT1 promoter resulted in slowed growth of H3.3 K27M patient-derived cells [140]. Additionally, H3K27me3 is elevated at *MHC1*, reducing MHC1 expression [122]. Furthermore, the loss of MHC1 expression has been linked to immune evasion in gliomas [146].

In summation, H3.1 and H3.3 K27M mutations are tumor-initiating mutations that cause the widespread loss of the transcriptionally repressive H3K27me3 mark. This loss of transcriptional repression activates genes that promote stemness and proliferation of the tumor cell. At the same time, there is very selective retention of K27me3 at a small number of tumor suppressor genes which allows unregulated tumor cell proliferation.

## 3. H3K27me3 in Ovarian Cancer

### 3.1. Epidemiology of Ovarian Cancer

Ovarian cancer accounts for approximately 13,000 deaths annually in the United States [147]. Among the various forms of ovarian cancer, epithelial ovarian cancer accounts for 90% of cases making it the most common type of ovarian cancer [148]. Unlike diffuse midline glioma, epithelial ovarian cancer occurs late in life, with the highest prevalence among patients in their 70s [148]. Furthermore, while the survival rate of epithelial ovarian cancer is better than that of diffuse midline glioma, 53% of women diagnosed with epithelial ovarian cancer will succumb to the disease within five years [148]. Epithelial ovarian cancer is a highly metastatic disease, with new tumors often occurring in the pelvic region or abdominal cavity [149].

### 3.2. Elevated H3K27me3 Is Linked to Ovarian Cancer Stage and Metastasis

**Figure 2:**
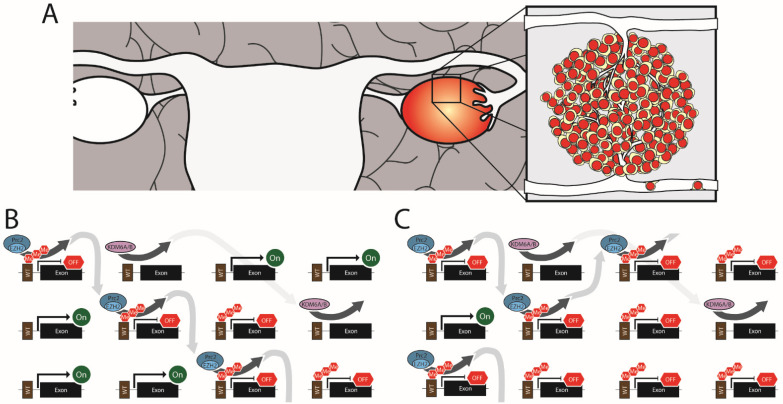
Epithelial Ovarian Cancer. (**A**) High-grade epithelial ovarian tumors arise from the ovaries but become metastatic. (**B**) Normal cells have a balance of PRC2 complexes triple methylating H3K27 and KDM6A/B demethylating H3K27. (**C**) In EZH2 overexpressing ovarian cancer, the system is overwhelmed by excess EZH2 driving excessive H3K27me3 levels and the silencing of genes.

The disruption of epigenetic gene regulation through altered H3K27me3 contributes to ovarian cancer. In ovarian cancer, these changes are most often the result of the overexpression of EZH2, leading to H3K27 hypermethylation (Figure 2). Among the different classes of ovarian tumors, EZH2 overexpression is most common in epithelial ovarian cancer, where it is observed in ~66% of primary tumors [92]. However, EZH2 overexpression has also been reported in mucinous epithelial ovarian cancer and non-epithelial ovarian cancers [150]. EZH2 levels in ovarian cancer correlate strongly with advanced cancer stage, metastasis, and tumor vascularization [92,94,150,151,152,153]. Whether or not this translates into higher mortality is uncertain. One study found that overexpression of EZH2 across all grades of epithelial ovarian cancer was strongly correlated (*p* < 0.001) with decreased overall survival (median survival of 2.5 years for EZH2 overexpression vs. 7.33 for EZH2 normal) [151]. However, a separate study that only looked at high-grade serous ovarian cancer found no correlation between survival and EZH2 expression [92]. One possible explanation for the discrepancy between these studies is that EZH2 expression may be a better predictor of tumor grade than mortality, so variations in EZH2 expression across tumors of the same grade are non-predictive of outcome. However, when all stages are lumped together, higher EZH2 expression in high-grade epithelial ovarian cancer correlates with mortality. Additionally, EZH2 overexpression is closely linked to metastatic ovarian cancer. When EZH2 mRNA levels were measured in patient tumors, 80% of metastatic ovarian cancers had significantly elevated levels compared to only 45% of non-metastatic cancer [152]. This association with metastatic disease probably accounts for some of the poor outcomes observed in patients with EZH2 high tumors.

### 3.3. EZH Drives Vascularization of Ovarian Cancer

Among epithelial ovarian cancers, 66% have elevated EZH2 expression in both the tumor cells and the associated epithelium [151]. To test the respective roles of EZH2 in the epithelium and the tumor cells, Lu et al. used human epithelial ovarian tumors in a xenograft mouse model that allowed targeted silencing of either vascular epithelium EZH2 (with mouse specific EZH2 siRNA) or tumor cells (with human specific EZH2 siRNA). Targeting the siRNA to the vascular epithelium resulted in less microvascular infiltration into the tumor [151]. This putative proangiogenic role for EZH2 in ovarian cancer is supported by in vitro studies where EZH2 knockdown reduced human umbilical vein endothelial cell (HUVEC) migration and endothelial tube formation in Matrigel [93]. On the other hand, when EZH2 was specifically silenced in the tumor cells there was a dramatic drop in lymph metastasis which suggests a pro-migration function of EZH2 overexpression [151].

### 3.4. EZH2 promotes Ovarian Tumor Cell Migration

Lu et al.’s observation that targeted EZH2 knockdown of ovarian tumor cells prevents metastatic formation in their mouse model may help explain the observation that EZH2 expression is elevated in metastatic human cancer [152]. The link to metastasis is also supported by cell biology as EZH2 siRNA knockdown in SKOV3 cells (an epithelial ovarian cancer patient line) significantly reduced cell migration and invasion in cell culture assays [92,151].

The pro-migratory effects of EZH2 overexpression on ovarian tumor cells involve the differential expression of multiple proteins. Rao et al. found that the genes for e-cadherin, TIMP2, TIMP3, and TIMP4 were all repressed in ovarian cancer tissues and that siRNA knockdown of EZH2 in an ovarian cancer cell line led to elevated expression of these genes [153]. Together these genes are involved in cell migration. E-cadherin functions in contact inhibition during cell proliferation. A loss of e-cadherin promotes cell migration and is a common feature of metastatic cancer (reviewed in [154]). Furthermore, TIMP proteins are inhibitors of the matrix metalloprotease family of proteins that digest the extracellular matrix. In epithelial ovarian cancer cell lines, EZH2 levels correlate inversely with TIMP2 levels via the EZH2 methylation of H3K27 on the *TIMP2* promoter [152,153]. Knockdown of EZH2 in an epithelial ovarian cancer cell line caused an increase in TIMP2 levels along with decreased levels of the active form of the matrix metalloproteases, MMP2 and MMP9. In in vitro assays, this loss of MMP2 and MMP9 inhibition coincided with reduced cell migration and invasion following EZH2 knockdown, which could be rescued with TIMP2 siRNA [152].

### 3.5. EZH2 Promotes Stemness and Loss of Cell Cycle Regulation in Ovarian Tumor Cells

EZH2 overexpression also causes loss of cell cycle control in epithelial ovarian cancers. Knockdown of EZH2 in epithelial ovarian cancer cell lines has been associated with the increased expression of many tumor suppressors involved in promoting cell-cycle arrest, including p14, p16, p21, p53, and p57 [150,155,156]. This suggests that EZH2 is necessary for epithelial ovarian cancer tumor survival to hold tumor suppressors in check.

Lastly, EZH2 overexpression promotes stemness in a sub-population of epithelial ovarian cancer cells. Comparing EZH2 staining to tumor differentiation by histology found a robust inverse correlation between EZH2 expression and differentiation with EZH2 overexpression in 36% of well-differentiated tumors, 83% of moderately differentiated tumors, and 93% of poorly differentiated tumors [150]. A potential explanation for this correlation is that EZH2 regulates DAB2IP expression through the methylation of H3 K27 at the DAB2IP promoter [157]. DAB2IP has Ras-GTPase activity and has been shown in various cancers to inhibit the cancer stem cell phenotype [158,159]. EZH2 knockdown reduced spheroid formation by 50% by an epithelial ovarian cancer cell line, suggesting that EZH2 functions in proliferation and stemness. Knockdown of DAB2IP and EZH2 together in these cells restored spheroid formation, demonstrating that suppression of DAB2IP expression by EZH2 promotes stemness in these cells [157].

## 4. Targeting H3K27me3 Pharmacologically

One approach to pharmacologically treat tumors with altered H3K27me3 levels would be to target each signaling pathway affected by the epigenetic change. Alternatively, all these pathways could be targeted at once by targeting epigenetics. As EZH2 overexpression is common in many cancers (Table 1), pharmacological inhibition has become a major area of interest in cancer research. Furthermore, in 2020, the Food and Drug Administration approved the first EZH2 inhibitor for use in the clinic, Tazverik (Tazemetostat, EPZ-6438). Tazverik is currently approved in the United States to treat lymphoma. The approval of Tazverick may just be the being as CliniclaTrials.gov listed 51 active trials into EZH2 inhibitors in cancer as of February 2022. Many of these studies are focused on lymphomas or leukemias, with only two trials in pediatric glioma and none in ovarian cancer. While the utilization of drugs targeting H3K27 methylation is not yet commonplace in the clinic for either of the two cancers highlighted in this review, there is evidence from the lab to suggest that such an approach holds promise.

Given the importance of EZH2 to late-stage metastatic ovarian cancer, EZH2 inhibition would appear to be a viable target for drug treatment; however, few small molecule studies have been undertaken in this tumor type. In one study, inhibition of EZH2 did not affect the viability of ovarian cancer stem cells in culture but showed a synergistic effect on reducing viability when combined with cisplatin [160]. However, a separate study found that EZH2 inhibition slowed ovarian cancer cell growth in culture in a dose-dependent manner. To test the efficacy of this approach in vivo, xenograft mice implanted with the ovarian cancer line, OVCAR3, were treated with cisplatin and an EZH2 inhibitor, GSK126, separately and in combination. While this mouse study showed no additive effects of cisplatin in combination with GSK126, GSK126 alone resulted in significantly smaller tumors after 38 days of treatment than untreated mice [157].

Interestingly, inhibiting EZH2 has also shown promise in the lab as a therapeutic for diffuse midline glioma, even though H3K27me3 levels are already significantly reduced. In a panel of diffuse midline glioma patient-derived lines, the EZH2 inhibitors, GSK343 and EPZ6438, slowed the proliferation of H3.3 K27M cell lines but not the H3 WT cells [126]. EPZ6438 significantly improved the survival of mice implanted with H3 K27M neuronal stem cells compared to untreated [161]. The thinking behind the efficacy of this approach is that blocking EZH2 removes the remaining H3K27me3, including that on the p16 gene. Thus, EZH2 inhibition kills the K27M cells by promoting p16 expression and inducing cell cycle arrest [126].

A significant challenge in treating DMG with small molecules is getting the small molecule past the intact blood–brain barrier. In the case of the EZH2 inhibitors, it was shown that a low membrane penetrance and a high affinity for efflux pumps leads to very poor brain penetrance for many EZH2 inhibitors that showed promise in cell culture [162].

Instead of further suppressing K27me3, finding a small molecule that can restore global H3K27me3 to the cells seems like a reasonable approach to treating DMG. The inhibition of the K27me3 demethylase, JMJD3, has shown promise. In diffuse midline glioma patient cells, the JMJD3 inhibitor, GSKJ4, increased K27me3 levels and inhibited colony formation only in those lines that harbored the K27M mutation; having little or no effect on H3.3 WT lines. Furthermore, GSKJ4 dramatically reduced tumor growth and improved survival in an H3.3 K27M xenograft mouse model [163].

As H3K27ac is elevated by the H3 K27M mutation [113,125,126,164], one pharmacological approach is to target the bromodomain and extra-terminal tail (BRET) proteins which act as readers for H3K27ac. When the BRET inhibitor, JQ1, was tested in culture, it selectively killed cells containing H3.1 or H3.3 K27M compared to H3 WT glioma lines [164]. Furthermore, RNA-Seq of these cells showed widespread repression of transcription with JQ1 in these lines, indicating a reversal of the gene activation caused by K27M. Finally, JQ1 treatment significantly reduced tumor size and significantly improved survival in an H3.3 K27M xenograft mouse model [164]. To our knowledge, no studies with these inhibitors have specifically looked at them in the context of EZH2 overexpressing ovarian cancer.

## 5. Conclusions

In summation, post-translational H3K27me3 modification is a primary epigenetic gene regulator. Furthermore, dysregulation of H3K27me3, by various mechanisms, is an essential contributing factor in many cancer types (Table 1). Yet, the changes in H3 K27 methylation during tumor formation and maturation are not universal across all cancer types, as we have illustrated here in the cases of diffuse midline glioma and epithelial ovarian cancer. In diffuse midline glioma, the global loss of the repressive H3K27me3 mark, paired with the very selective retention of that mark at a small number of tumor suppressor genes, is the driving event in gliomagenesis. Yet, in epithelial ovarian cancer, the changes in H3K27me3 appear at a late stage of the tumor’s progression.

It is important to point out that the EZH2 complex may have functions beyond H3K27 methylation, although they are poorly understood. For instance, EZH2 can directly methylation the RORα receptor [165]. Furthermore, EZH2 is both cytoplasmic and nuclear, and in the cytoplasm, it may play direct roles in actin polymerization and cell signaling [166]. These non-canonical roles of EZH2 are not well characterized and if they play any role in cancer is currently unknown.

While the link between epigenetic gene regulation by H3 and tumor formation in diffuse midline glioma and epithelial ovarian cancer is well established, it is crucial to keep in mind that histones function in various other cellular processes besides just transcription regulation. For example, H3 post-translational modifications are involved in DNA damage response [167], telomere maintenance [168], and regulation of cell ploidy [169,170]. These alternative functions of H3 PTMs are generally less well understood than the epigenetic functions and deserve more attention from the cancer research community. A better understanding of how common histone PTMs function in these non-canonical roles could tremendously impact our understanding of cancer. For example, there seems to be a disconnect between the degree of differences in patient outcome between the H3.1 K27M and H3.3 K27M diffuse midline gliomas, even though transcriptome data shows the epigenetic effects of these tumors are highly similar. What accounts for the differences in patient outcomes between these two groups is unclear. Some of the differences in outcome are probably attributable to the distinct co-mutations. However, there may be additional functional changes to H3’s non-epigenetic function that are disrupted by the K27M mutations, and this possibility has not been adequately explored.

## Data Availability

Not applicable.
